# Can staff and patient perspectives on hospital safety predict harm-free care? An analysis of staff and patient survey data and routinely collected outcomes

**DOI:** 10.1136/bmjqs-2014-003691

**Published:** 2015-04-10

**Authors:** Rebecca Lawton, Jane Kathryn O'Hara, Laura Sheard, Caroline Reynolds, Kim Cocks, Gerry Armitage, John Wright

**Affiliations:** 1 Institute of Psychological Sciences, University of Leeds, Leeds, UK; 2 Department of Quality and Safety Research, Bradford Institute for Health Research, Bradford, UK; 3 Department of Health Sciences, York Trials Unit, York, UK; 4 School of Health, University of Bradford, Bradford, UK; 5 Department of Epidemiology and Public Health, Royal Infirmary Bradford, Bradford, UK

**Keywords:** Patient safety, Health services research, Surveys, Safety culture, Patient-centred care

## Abstract

**Background:**

Patients have the potential to provide feedback on the safety of their care. Recently, tools have been developed that ask patients to provide feedback on those factors that are known to contribute to safety, therefore providing information that can be used proactively to manage safety in hospitals. The aim of this study was to investigate whether the safety information provided by patients is different from that provided by staff and whether it is related to safety outcomes.

**Method:**

Data were collected from 33 hospital wards across 3 acute hospital Trusts in the UK. Staff on these wards were asked to complete the four outcome measures of the Hospital Survey of Patient Safety Culture, while patients were asked to complete the Patient Measure of Safety and the friends and family test. We also collated publicly reported safety outcome data for ‘harm-free care’ on each ward. This patient safety thermometer measure is used in the UK NHS to record the percentage of patients on a single day of each month on every ward who have received harm-free care (ie, no pressure ulcers, falls, urinary tract infections and hospital acquired new venous thromboembolisms). These data were used to address questions about the relationship between measures and the extent to which patient and staff perceptions of safety predict safety outcomes.

**Results:**

The friends and family test, a single item measure of patient experience was associated with patients’ perceptions of safety, but was not associated with safety outcomes. Staff responses to the patient safety culture survey were not significantly correlated with patient responses to the patient measure of safety, but both independently predicted safety outcomes. The regression models showed that staff perceptions (adjusted r^2^=0.39) and patient perceptions (adjusted r^2^=0.30) of safety independently predicted safety outcomes. When entered together both measures accounted for 49% of the variance in safety outcomes (adjusted r^2^=0.49), suggesting that there is overlap but some unique variance is also explained by these two measures. Based on responses to the Patient Measure of Safety it was also possible to identify differences between the acute Hospital Trusts.

**Discussion:**

The findings suggest that although the views of patients and staff predict some overlapping variance in patient safety outcomes, both also offer a unique perspective on patient safety, contributing independently to the prediction of safety outcomes. These findings suggest that feedback from patients about the safety of the care that they receive can be used, in addition to data from staff to drive safety improvements in healthcare.

**Trial registration number:**

ISRCTN07689702.

## Introduction

A recent report on the measurement of safety^1^ concluded that there is no ‘single’ measure of patient safety, but that monitoring and measurement needs to be backward-looking, present-looking and forward-looking. That is, we need to know about and learn from past harm, understand what is going on in real time and anticipate future harm. The authors concluded that while healthcare organisations have grappled with the first of these (through incident reporting systems, root cause analysis and more recently the patient safety thermometer) and have made some progress with the second (eg, via early warning scores), the third remains somewhat elusive.

Moreover, safety measurement has relied almost exclusively on eliciting information from staff (directly, eg, through incident reports and questionnaire measures, or indirectly, eg, through case note review) or requiring them to record information (eg, on the number of falls or pressure sores). More recently, however, following reports in the UK such as Francis;^2^ Keogh^3^ and Berwick^4^, commentators have emphasised the importance of patients as the ‘smoke detectors’ for safety.^5^ These ideas have a growing evidence base with increasing numbers of international studies reporting that patients are able to make a valuable contribution to our understanding of safety.^6–8^ Indeed, patients do have a voice, at least in some countries, and there are a growing number of measures that ask patients to report on their experience of care in the UK (eg, the Picker survey^9^ and the friends and family test^10^) and elsewhere, (eg, Hospital Consumer Assessment of Healthcare Providers and Systems (HCAHPS)^11^ in the USA and the Australian Patient Experience Questionnaire (2014;^12^ see Castle *et al*,^13^ for a review). Rarely, however, are patients asked about the safety of their care or their care environment and to date most studies have asked patients to report on outcomes (patient safety incidents, eg, The National Danish Survey of Patient Experiences^14^) rather than on the factors that might represent failures in structures and processes (ie, systems of care). An exception to this is the Patient Measure of Safety (PMOS^15^ ^16^) developed by the Yorkshire Quality and Safety Research Group. PMOS provides a proactive assessment of the organisational and local system factors known to contribute to patient safety incidents in hospitals. In other words, it is a forward-looking measurement instrument that provides information, based on patient perceptions, about how the ward/hospital is performing in a number of safety critical domains including communication and team work, access to resources, organisation and care planning, roles and responsibilities, ward type and layout, information flow, staff training, delays and equipment; those factors known to contribute to patient safety incidents.^17^ The PMOS tool has been extensively piloted and found to be reliable, valid and well received by patients.^15^ ^16^ What is yet untested is the extent to which these tools provide the same or a different perspective on safety from more traditional patient safety measurement tools that are based on staff perceptions of safety or safety outcomes (as reported by staff).

The aims of this study were to investigate (A) the extent to which the staff and patient measures of safety correlate together and with the percentage of patients receiving harm-free care (% harm-free care) (patient safety thermometer data) (b) to explore the extent to which the staff and patient perspective on safety provide information that can explain variation in % harm-free care and (c) describe variation in these measures in three acute hospital Trusts.

## Methods

### Participants

Participants were patients in one of 33 hospital wards, across five acute hospitals in three acute hospital Trusts in the Yorkshire and Humber region of the UK during May–July 2013. Patients who were deemed to have the capacity to respond to the measures (following discussion with the nurse in charge of the ward) and who had been admitted to hospital a minimum of 4 h previously, were approached by one of a team of six research nurses and invited to take part in the study. The aim was to collect data from a minimum of 15 patients per ward, recognising that in some wards (eg, elderly medicine) patient throughput would be much slower than in others (medical admissions unit).

Staff on each of the participating wards were also asked to complete the four outcome measures of the Agency for Healthcare Research and Quality (AHRQ) Hospital Survey of Patient Safety Culture^18^ (hereafter referred to as the HSOPSC) patient safety culture tool.

### Measures

Two measures of patient experience and safety and one measure of staff safety culture were used

1. Patient perceptions of safety. The PMOS includes 44 items measuring 9 constructs: communication and teamwork (9 items); organisation and care planning (5 items); ward type and layout (11 items); equipment (2 items); roles and responsibility (4 items); access to resources (4 items); information flow (3 items); staff training (2 items) and delays (2 items). A score was calculated for each construct by taking a mean of the responses to all those items making up the domain. Where scores were not available for at least two items within a construct, the domain score was coded as missing data. The questionnaire is supplied as an online supplementary file here because the wording of some items differs from the measure previously published, due to changes following further piloting. Items are presented as statements with which patients are asked the extent to which they agree or disagree on a five-point Likert scale, with a higher score representing stronger agreement. Negatively worded items are recoded. The PMOS scale showed strong internal reliability (α=0.93). A mean of the construct scores was calculated to give a PMOS mean score between 1 and 5. Where scores were not available for one or more of the nine constructs, the PMOS mean score was coded as missing.
2. Patient experience. The friends and family test was also completed by each patient. This asks “How likely are you to recommend this ward to your friends and family if they needed similar care or treatment?” Responses are provided on a six-point scale from ‘extremely unlikely’ to ‘extremely likely’. As part of routine data collection this question is asked of patients at discharge or no more than 48 h afterwards. In this case, we asked patients to complete the measure at the same time as the PMOS which was always prior to discharge.
3. Staff safety culture. Nurses on participating wards were asked to complete the four outcome measures from the HSOPSC^18^. These are (1) perceptions of patient safety (four items, 1=‘strongly disagree’ to 5=‘strongly agree’); (2) patient safety grade (one item, 1=‘failing’ through to 5=‘excellent’); (3) frequency of events reported (three items, 1=‘never’ through to 5=‘always’); and (4) number of events reported (one item, categorical response options ranging from 1=‘no event reports’ through to 6=‘21 event reports or more’). The brief questionnaire was devised to promote a high response rate by minimising the burden on staff time.

### Patient safety outcomes

Patient safety outcome data is publicly available for all NHS Trusts in England. The NHS Safety Thermometer (http://www.hscic.gov.uk/thermometer)^19^ asks staff on hospital wards to record the presence or absence of four harms on a single day each month: pressure ulcers, falls, urinary tract infections in patients with a catheter and new venous thromboembolisms. Based on these data a score is computed for the percentage of patient assessments which showed none of the harms and therefore represents ‘harm-free’ care. We downloaded the data for all wards in the three participating acute hospital Trusts. Data were downloaded for each of the 4 months in which PMOS and staff data were collected at our participating hospitals to generate a ‘harm-free’ score and dividing by 4 (months) we created a mean ‘harm-free’ care score. This score is therefore a proportion (%) of patients on each ward who are recorded as not having been exposed to any of the four harms, based on the mean of 4 months data.

### Procedure for data collection

Patients were approached by a research nurse and provided with a brief synopsis of the purpose of the study. Interested patients were asked to read the information sheet and give their informed consent to participate. Patients were asked if they wanted to complete the questions directly or whether they wanted the research nurse to ask them the questions and then record their responses on the tablet computer (19% chose to complete the questionnaire themselves). The friends and family test was also completed by all patients as part of the collection of other data. Where problems or incidents were reported that were identified by the research staff as safety critical and requiring an immediate response, the patient was informed that the information would be passed on to the person in charge. We aimed to recruit 25 patients per ward (with a minimum of 15 patients) over a 4 week period. This number was a balance between achieving sufficient patients per ward to reasonably estimate parameters while capturing data within a short enough period so that substantial ward changes in that time frame were unlikely.

Staff culture questionnaires were prepared for members of staff employed on each ward using staff lists collected from the ward prior to the recruitment period. When research nurses began recruitment of patients they met with the ward sister/manager and asked him/her to distribute the questionnaires to staff and encourage completion. Staff returned questionnaires using drop boxes which were collected from the ward no longer than a month after patient recruitment finished. This was to ensure that feedback from staff and patients was collected during the same time period. To allow for the collection of data from staff who were not employed by a particular ward/unit, for example, doctors, five blank questionnaires were also made available for each ward and the sister was asked to ensure these were all distributed. All questionnaires were labelled with a code that allowed identification of the trust and ward, but not the individual.

The aim was to recruit a minimum of 50% of the staff on each ward. Where this target was not reached at the end of the patient data collection period, research nurses visited the wards on up to two further occasions to encourage questionnaire completion (blank copies were made available if necessary).

### Analysis

Frequencies and descriptive summaries were run to explore the numbers and types of patients and staff on each ward and to check for any anomalous values and explore patterns of responding across wards.

The unit of analysis here was ward. All measurements from staff or patients were averaged at the ward level before entering into the data file. The harm-free care score was only available at ward level. Pearson correlations were run to assess the association between harm-free care score (averaged score across the 4 months of data collection), PMOS (total score for each ward) and the four patient safety outcomes from the HSOPSC Hospital Survey of Patient Safety. Correlation coefficients of 0.1 were considered small; 0.3 moderate and 0.5 and above large.^20^ All variables are coded such that a positive score equals good levels of safety, so that we anticipated positive correlations among all variables. There was one exception to this. Positive correlations were predicted for all variables with the exception of the number of events reported. Higher number of events reported can indicate either good or bad levels of safety (ie, it could be that staff are doing a good job of reporting all errors (good) or it could be that there are a lot of errors to report (bad)). Scatterplots (see online resources) were run to assess whether relationships between variables were linear and whether particular wards represented outliers with respect to patterns of correlations. Spearman's rank correlations were also computed to ensure that the pattern of findings were the same if assumptions of linearity were not met.

Linear regressions were computed to assess the predictive value of the different measures of safety. Only those variables that demonstrated univariate correlations with the % harm-free care score (dependent variable) were entered into the regression. In each regression ward size (number of patients treated on the ward), type (medical or surgical) and average age of patients treated on the ward were first entered into the model as these represent the minimisation factors in our sampling strategy. The first regression model tests the amount of variance accounted for when only staff measures of safety (independent variable 1) are regressed onto % harm-free care. The second model investigates the variance accounted for when only the patient measures (PMOS; independent variable 2) is included in the model. The final model investigates the additional variance that is accounted for (r^2^ change) by the PMOS score having already accounted for the staff measures of safety in the model.

Although ward was the unit of analysis there is further clustering at the Trust level but with only three Trusts we could not run a multilevel model accounting for this. A MANOVA was run to compare Trusts across the four measures and to identify any differences in safety scores across Trusts.

## Results

Data were collected from 822 patients and 648 members of staff. The number and type of patients recruited varied across wards (see table 1). Recruitment was at the planned level (with a mean of 25 patients) for all but three of the participating wards. Response rates were high, above 80% on all but three wards and only falling below 70% on one ward. Those wards (1, 11 and 25) where we did not reach the minimum target of 15 respondents were elderly wards with a very slow turnover of patients (wards 1 and 25) or had a large number of patients who were unable to consent due to a lack of capacity, or severity of illness (ward 11). A response rate of 50% or above was achieved for the HSOPSC outcomes in Trust A and Trust B, but was poorer in Trust C (the largest of our three participating Trusts). All wards in all Trusts were nevertheless included in the analyses.

**Table 1:** Response rate for patient and staff surveys and demographic characteristics for patients recruited to the study

	Patient response rate n (%)	Mean age	% Female	% White British	Staff response rate n (%)	% nursing
Trust A						
Ward 1	14 (82)	73	14	100	20 (69)	45
Ward 2	35 (92)	55	0	97	25 (71)	60
Ward 3	35 (80)	50	71	100	19 (53)	47
Ward 4	26 (93)	72	54	92	21 (66)	67
Ward 5	30 (97)	60	37	100	30 (73)	67
Ward 6	31 (89)	68	100	100	25 (68)	36
Ward 7	18 (78)	56	61	100	17 (74)	71
Ward 8	16 (80)	66	67	100	26 (58)	46
Trust B						
Ward 9	26 (84)	58	35	85	20 (51)	55
Ward 10	34 (89)	66	59	91	24 (92)	58
Ward 11	14 (54)	69	43	100	18 (55)	67
Ward 12	32 (100)	59	31	94	15 (63)	47
Ward 13	29 (100)	60	69	90	15 (50)	40
Ward 14	22 (92)	60	48	86	17 (61)	53
Ward 15	18 (78)	61	83	94	19 (49)	53
Ward 16	26 (90)	62	31	88	26 (49)	62
Ward 17	27 (96)	52	100	88	27 (96)	70
Ward 18	25 (100)	63	52	96	25 (54)	44
Trust C						
Ward 19	18 (82)	64	29	100	15 (56)	60
Ward 20	32 (84)	66	0	90	17 (55)	59
Ward 21	30 (97)	47	30	87	28 (54)	57
Ward 22	29 (94)	51	45	86	13 (46)	38
Ward 23	29 (91)	51	3	97	8 (36)	63
Ward 24	29 (97)	62	72	96	10 (38)	30
Ward 25	8 (89)	86	100	100	20 (65)	40
Ward 26	22 (76)	67	38	91	11 (31)	45
Ward 27	18 (90)	60	0	88	16 (67)	38
Ward 28	20 (100)	57	100	85	15 (52)	53
Ward 29	16 (94)	76	50	88	19 (46)	58
Ward 30	27 (96)	62	93	96	22 (50)	55
Ward 31	29 (91)	53	3	86	24 (59)	67
Ward 32	32 (84)	56	47	97	22 (61)	45
Ward 33	25 (89)	61	48	92	19 (53)	42

### Correlation of different measures of the quality of care

The distributions for each of the seven variables to be correlated appeared normal and skew and kurtosis values for all variables were below 1, with the exception of frequency of events reported where values were slightly outside this range (−1.17 and 1.22, respectively). Thus the data were treated as normal and Pearson's correlations were run.^i^ The correlations between the four types of data (PMOS, harm-free care score, the four staff safety culture (HSOPSC) outcomes and friends and family test) are shown below in table 2. Scatterplots showing the distribution of variables and the patterns of associations between variables are shown in online supplementary figure S1.

**Table 2:** Mean (SD) for safety measures and correlations between measures (N=33 wards)

	Mean (SD)	% harm-free care	Friends and family test	PMOS mean score	HSOPSC—perceptions of safety	HSOPSC—patient safety grade	HSOPSC—frequency of events	HSOPSC number of events reported
% harm-free care (0–100)	91.61 (5.97)							
Friends and family test (1–6)	4.38 (0.25)	0.29						
PMOS mean score (0–5)	3.84 (0.17)	0.58***	0.69***					
HSOPSC—perceptions of safety	3.54 (0.39)	0.48**	-0.05	0.25				
HSOPSC—patient safety grade	3.80 (0.44)	0.54***	0.11	0.30	0.91***			
HSOPSC—frequency of events	4.04 (0.33	0.04	0.12	0.03	0.48**	0.56**		
HSOPSC—number of events reported	2.39 (0.52)	−0.41*	−0.39*	−0.43*	−0.47**	−0.44*	−0.36*	

*p<0.05; ** p<0.01; *** p<0.001.

% harm-free care, percentage of patients receiving harm-free care; HSOPSC, Hospital Survey of Patient Safety Culture; PMOS, Patient Measure of Safety.

The correlations in table 2 show that while the friends and family test score is correlated with the PMOS, it is not significantly correlated with either the staff safety culture (HSOPSC) or the % harm-free care reported in participating wards. The friends and family test was therefore excluded from the regression analysis. The strongest correlations were between the PMOS and the % harm-free care (r=0.58) score and between the HSOPSC patient safety grade and the % harm-free care score (r=0.54). Perceptions of patient safety (0.48) and number of patient safety events reported (−0.41) were also significantly correlated with % harm-free care. The latter negative correlation indicates that as the number of patient safety events reported by staff increased, the % harm-free care decreased. The frequency of reporting of events was not associated with harm-free care and therefore will not be included in the subsequent analysis. Interesting too was the lack of a significant correlation between the perceptions of patient safety, the patient safety grade and the PMOS score. However, the number of events reported by staff did show a significant negative correlation (r=−0.43) such that the more safety events reported by staff, the lower the PMOS score. The very high correlations between the HSOPSC patient safety grade and perceptions of safety (r=0.91) indicated that these two scales were measuring the same thing and therefore to avoid multicollinearity, only the patient safety grade score was entered in the regression analysis.

### Factors associated with safety outcome (harm-free care score)

Three separate regressions were computed. In each case, ward characteristics were entered on the first step to control for any variation in patient safety outcomes due to factors that might be anticipated to co-vary with safety outcomes (ward size, average age of patients and whether the ward was surgical or medical). Together the ward characteristics accounted for 15% (r^2^=0.15) of the variance and this model was not statistically significant (p=0.06).

#### Regression model 1

Staff measures of safety (patient safety grade and number of events reported in the last 12 months), were entered on the second step and the regression model was significant (F (5,26)=5.04, p<0.01) and the adjusted r^2^ was 0.39. The standardised regression coefficient was significantly different from zero for patient safety grade (β=0.43, p<0.05) but not for number of events reported (β=−0.31, p=0.08).

#### Regression model 2

PMOS was entered on step 2 and the regression model was significant (F (4, 27)=4.39, p<0.01) and the adjusted r^2^ was 0.30. The standardised regression coefficient for PMOS was significant in the model (β=0.50, p<0.05).

#### Regression model 3

In this model, the staff measures of safety were entered on step 2, followed by the patient measures of safety on step 3. See model 1 above for the results of step 2. On step 3, the adjusted r^2^ was 0.49 and the model was statistically significant (F(6,25)=6.02, p<0.001). Patient safety grade (β=0.42, p<0.01), PMOS (β=0.40, p<0.05) and average age of patients on the ward (β=−0.38, p<0.05) were significant predictors of % harm-free care, such that more positive staff grades of safety, more positive patient perceptions of safety and wards with younger patients were all associated with less harm to patients.

#### Variation in scores between hospitals

Table 3 shows the means and SDs for each of the participating NHS Trusts on the four types of measure. The friends and family test is consistently high and positive, across Trusts. The scores on the PMOS and the HSOPC were lower and less positive overall. The % harm-free care scores indicate that in Trust A, 7.00% of patients are harmed, in Trust B this is lower at 5.66% and highest in Trust C at 11.13%. The lowest performing Trust across all four of the measures was Trust C.

**Table 3:** Means (SD) for each Trust on each of the patient safety measures

	Trust A	Trust B	Trust C	F (sig)	p Value
Friends and family test	4.48 (0.23)	4.43 (0.25)	4.30 (0.24)	1.53	0.23
PMOS score	3.91 (0.16)	3.93 (0.08)	3.74 (0.19)	5.55	0.009
HSOPSC total	3.93 (0.33)	3.82 (0.27)	3.75 (0.35)	0.81	0.46
Harm-free care score	93.00 (6.01)	94.34 (2.95)	88.87 (6.68)	3.10	0.06

HSOPSC, Hospital Survey of Patient Safety Culture; PMOS, Patient Measure of Safety.

A multivariate analysis of variance was used to investigate the differences in scores across the four measures for the three Trusts from which we collected data. There was a main effect of Trust (F(5,25)=2.14, p<0.05). Inspection of the univariate differences revealed that while the friends and family test and the HSOPSC outcomes did not differ significantly, the PMOS scores did demonstrate a significant difference between Trusts. The harm-free care score was close to significance. Bonferroni pairwise comparisons revealed that only Trust B and Trust C differed significantly (p=0.009) on the PMOS measure, with Trust A and Trust C showing differences, but which did not reach statistical significance (p=0.06).

## Discussion

Patient safety is a multifaceted and complex concept which may be assessed differently by staff and patients.^16^ Indeed there is some empirical evidence which suggests that patients might offer a different perspective on safety from staff.^21^ ^22^

We found that two measures showed strong correlations with patient safety outcomes as measured by %harm-free care. These were the staff measure of safety culture (HSOPSC outcomes) and the PMOS. The regression models showed that staff perceptions (adjusted r^2^=0.39) and patient perceptions (adjusted r^2^=0.30) of safety independently predicted safety outcomes. When entered together both measures accounted for 49% of the variance in safety outcomes (adjusted r^2^=0.49), suggesting that there is some overlap but also a unique variance explained by these two measures. In other words, what patients tell us about the safety of their care may partially overlap with staff perceptions, but patients tell us something that helps us to understand more about the safety outcomes on hospital wards. The friends and family test, which is purported to measure patient experience of care was significantly correlated with the PMOS, which measures patient perceptions of the safety of care. However the friends and family test did not show any relationship with patient safety outcomes or staff measures of safety. This suggests that while overall patient experience and patient experience of safety may overlap, only the latter, more specific, measure provides a potentially useful measure of the safety of care.

It could be argued that in completing questionnaires about their perceptions of safety, staff will be influenced by recent harm-free care scores, raising the possibility that the relationship between these two variables is bidirectional. The same is unlikely to be true for patient scores of safety. Some, but not all wards, make their harm-free care scores publicly available through wall displays in wards. However, with nothing in the way of comparator data, patients are unlikely to know whether the scores are positive or not and therefore the impact on their ratings of safety are unlikely to be strong.

The PMOS measure includes a large number of items and therefore is somewhat onerous to complete, taking approximately 10–15 min per patient. However, it has a number of advantages that make it an important addition to the battery of measurement tools for patient safety. First, asking patients, rather than staff to complete surveys, reduces the burden on staff. Second, the 44-item PMOS questionnaire provides feedback on the general perceptions of patients about safety, and offers unique information about the performance of a ward or unit in nine domains of safety.^15^ ^16^ This information can be used by wards to inform and plan local improvements.

A key attribute for any measure of quality and safety is that it should be able to describe opportunities for improvement. Many patient measures (eg, patient satisfaction and the friends and family test) suffer from a ceiling effect where patients are consistently positive in their responses. In this study we explored the extent to which measures might discriminate across Trusts. The findings here revealed that the PMOS scores do vary across Trusts and that it is possible to identify significant differences in patient perceptions of safety that might provide an important and proactive measure of safety at an organisational level. In contrast measures of patient experience and staff culture were not significantly different across Trusts. However, caution should be taken in interpretation given that safety outcomes are strongly influenced by case mix (eg, pressure sores and falls are more common on elderly care wards).

The potential value of the PMOS tool as a proactive diagnostic tool should not be underestimated. While measures such as the friends and family test are parsimonious and may, with sufficiently high response rates, offer a means of comparing healthcare organisations or identifying problems, they provide information that is of little value to healthcare providers in delivering improvement. The collection of data without any real purpose is rife in the NHS. Policy makers should think carefully about what a measure offers before requiring providers to spend time and resources collecting data and providers should embrace measures which provide them with valuable information about what they can do to improve. We believe PMOS does just that.

### Limitations

The 50% target response rate to the staff culture survey was not achieved on some wards, particularly in Trust C. Although research staff administered and distributed the questionnaires, the nature of data collection meant there was a reliance on ward managers/sisters to encourage their staff to complete the survey. Encouragement was often related to how engaged the senior ward staff were with the study and also their style of leadership. Some ward managers were more proactive than others and personally handed them out, some did nothing at all to encourage completion. After two further visits by research nurses to wards with low response rates, the research team agreed that data collection should cease to ensure that the patient and staff feedback was elicited over the same period of time. Moreover, we achieved good response rates for the majority of wards and data were analysed at ward level so variation in response rates is very unlikely to explain the relationships between variables.

We did not recruit patients who either lacked capacity or were too ill to participate, to complete PMOS. While this is ethically justifiable, we may have also inadvertently excluded those patients who, through experiencing a broader range of treatment related interventions and sometimes for a longer time period, might have provided an important perspective on organisational safety. Our research nurses were aware of this and were careful to return to patients who they were not able to recruit on day 1 to check whether they were able to consent on a later day (eg, when they were 2 days postop or if they were having a more lucid episode). It may also be appropriate, in future, to elicit the views of these groups by asking friends or relatives to complete PMOS on the patient's behalf. However, further research will need to be undertaken to assess the extent to which the views of friends and relatives reflect those of the patients, being on the wards, as they generally are, for a limited time and at particular times of the day.

In this study, data were collected by dedicated research nurses who have been working on this project since the empirical phase began. They have developed a specific repertoire of skills that allow them to engage with patients about their safety and have received training in the theory of patient safety. Such preparation would have some resource implications for an organisation who might wish to adopt the interventions. It will be necessary to explore alternative and potentially more efficient approaches to data collection, for example, via hospital volunteers or undergraduate medical and nursing students.

### Further research

It is important to note that the harm-free care score assesses a limited number of harms. For example, it does not assess medication errors, other infections, misdiagnosis, etc. It is not possible to conclude that the same pattern of relationships will exist for other safety outcome measures. Further exploration of these relationships for other safety outcome measures is necessary.

PMOS has been designed for the acute care sector. However, the design has a number of principles that have the potential for transferability to other sectors. It is entirely feasible therefore that equivalent measures could be developed for primary, community and mental healthcare organisations albeit with some modification to the domains and the questions used to assess them within the questionnaire.

The PMOS questionnaire, as well as providing a quantitative measure of the safety of the care environment through the eyes of patients, was developed primarily as a diagnostic tool. Wards/departments can use the scores on the nine domains to proactively identify local areas of strength and weakness and to plan for safety improvements using this information. A cluster randomised study is currently underway to evaluate the impact on safety outcomes of using the PMOS tool in this way. Further research will be needed to understand whether approaches such as this represent cost-effective solutions to improving safety in hospitals and the extent to which this varies as a function of different data collection methods.

## Conclusion

This study showed that a patient measure of ward safety and a staff culture measure were associated with the level of harm-free care. PMOS provides a distinctive patient-centred measure of patient safety that has the potential to inform safety improvement actions appropriate to individual wards.

## Supplementary Material

Web supplement

Web figure

## Footnotes

i Spearman's rank correlations were also computed, but the patterns and significance of the relationships did not change when using this method, although the sizes of the correlations were consistently more conservative.
