# Supplementary material for: Can staff and patient perspectives on hospital safety predict harm-free care? An analysis of staff and patient survey data and routinely collected outcomes
Source: BMJ Qual Saf. 2015 Apr 10;24(6):369–76. doi: 10.1136/bmjqs-2014-003691 (PMC4453491; doi:10.1136/bmjqs-2014-003691)
Supplement: Web supplement [file bmjqs-2014-003691-s1.pdf]

PMOS Questionnaire items		
**Question Number**	**Question Text**	**Domain**
1	I was always treated with dignity and respect	None
2	I knew who to go to if I needed to ask a question	Organisation and care planning
3	The drugs I have been prescribed were always available in hospital	Organisation and care planning
4	I got answers to all the questions I had about my care	Communication and teamworking
5	Staff were always able to get advice from other teams about my care if needed	Communication and teamworking
6	A doctor changed my plan of care and other staff didn't know about it	Organisation and care planning
7	After a shift change staff did not appear to know important information about my care	Information flow
8	I knew what the different roles of the people caring for me were	Staff roles and responsibilities
9	On at least one occasion a member of staff was not able to use the necessary equipment	Staff training
10	My treatment/ procedure/ operation did not always happen on time	Delays
11	The following aspects of the ward made it difficult for staff to do their jobs: Position of nurses' station	Ward type and layout
12	The following aspects of the ward made it difficult for staff to do their jobs: Lighting levels	Ward type and layout
13	The following aspects of the ward made it difficult for staff to do their jobs: Clutter & untidiness	Ward type and layout
14	The following aspects of the ward made it difficult for staff to do their jobs: Lack of	Ward type and layout

	Space	
15	I was on a ward that was not able to deal with my treatment needs	Ward type and layout
16	Staff were prompt in answering my buzzer	Ward type and layout
17	It was clear who was in charge of the staff	Staff roles and responsibilities
18	Sometimes there was no-one available to deal with aspects of my care	Communication and teamworking
19	On at least one occasion a member of staff was not able to carry out a task that they should have been able to do	Staff training
20	The following aspects of the ward made it uncomfortable for me: Noise levels	Ward type and layout
21	The following aspects of the ward made it uncomfortable for me: Lighting levels	Ward type and layout
22	The following aspects of the ward made it uncomfortable for me: Temperature	Ward type and layout
23	The following aspects of the ward made it uncomfortable for me: Poor cleanliness	Ward type and layout
24	The following aspects of the ward made it uncomfortable for me: Lack of space	Ward type and layout
25	The following aspects of the ward made it uncomfortable for me: Other	
26	I felt that the attitude of staff towards me was poor	Communication and teamworking
27	I knew which consultant was in charge of my care	Staff roles and responsibilities
28	Staff always seemed to know what they were meant to be doing	Communication and teamworking
29	There were enough staff on the ward to get things done on time	Delays
30	Staff gave me different information about my care	Organisation and care planning

31	Staff/patients waited a long time for porters to arrive	Access to resources
32	Staff did not work together as a team here	Communication and teamworking
33	There was equipment that staff found difficult to use (e.g. monitoring equipment, beds, hoists)	Equipment (design and functioning)
34	I have needed treatment and there has been no-one available who was trained to do it	Organisation and care planning
35	Staff were kept waiting for my test results	Information flow
36	Nurses were always able to get help from other staff when they asked for it	Access to resources
37	Equipment needed for my care was always working properly	Equipment (design and functioning)
38	I always knew which nurse was responsible for my care	Staff roles and responsibilities
39	Equipment and supplies were not always available when needed (e.g. hoists, bed pans, drugs)	Access to resources
40	Staff always agreed about my treatment/care	Communication and teamworking
41	I always felt staff listened to me about my concerns	Communication and teamworking
42	Staff seemed to struggle to get help when they needed it	Access to resources
43	When staff talked about my care with others the information they shared was correct	Communication and teamworking
44	Information about me that my health care team needed was always available (e.g. drug charts, medical notes, test results)	Information flow
