# Supplementary material for: Can staff and patient perspectives on hospital safety predict harm-free care? An analysis of staff and patient survey data and routinely collected outcomes
Source: BMJ Qual Saf. 2015 Apr 10;24(6):369–76. doi: 10.1136/bmjqs-2014-003691 (PMC4453491; doi:10.1136/bmjqs-2014-003691)

Figure 1:Scatterplots and distributions of responses for friends and family test (FFT), patient measure of safety (PMOS), the outcome measures of the hospital survey of patient safety survey (HSOPSC1: perceptions of patient safety; HSOPSC2: frequency of events reported; HSOPSC3: number of events reported in the last 12 months; HSOPSC4: patient safety grade) and %harm-free care.

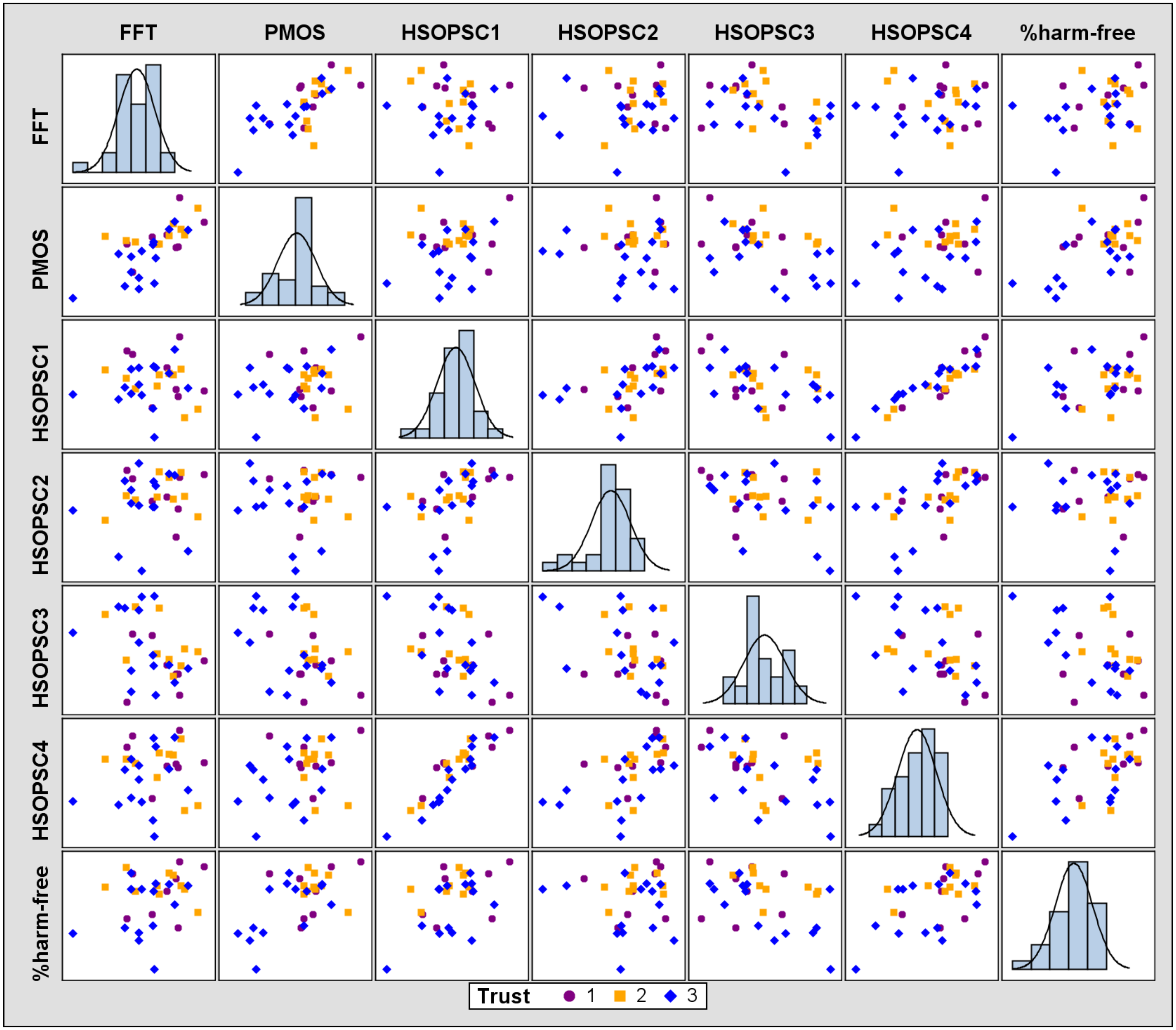

Supplement: Web figure [file bmjqs-2014-003691-s2.pdf]
